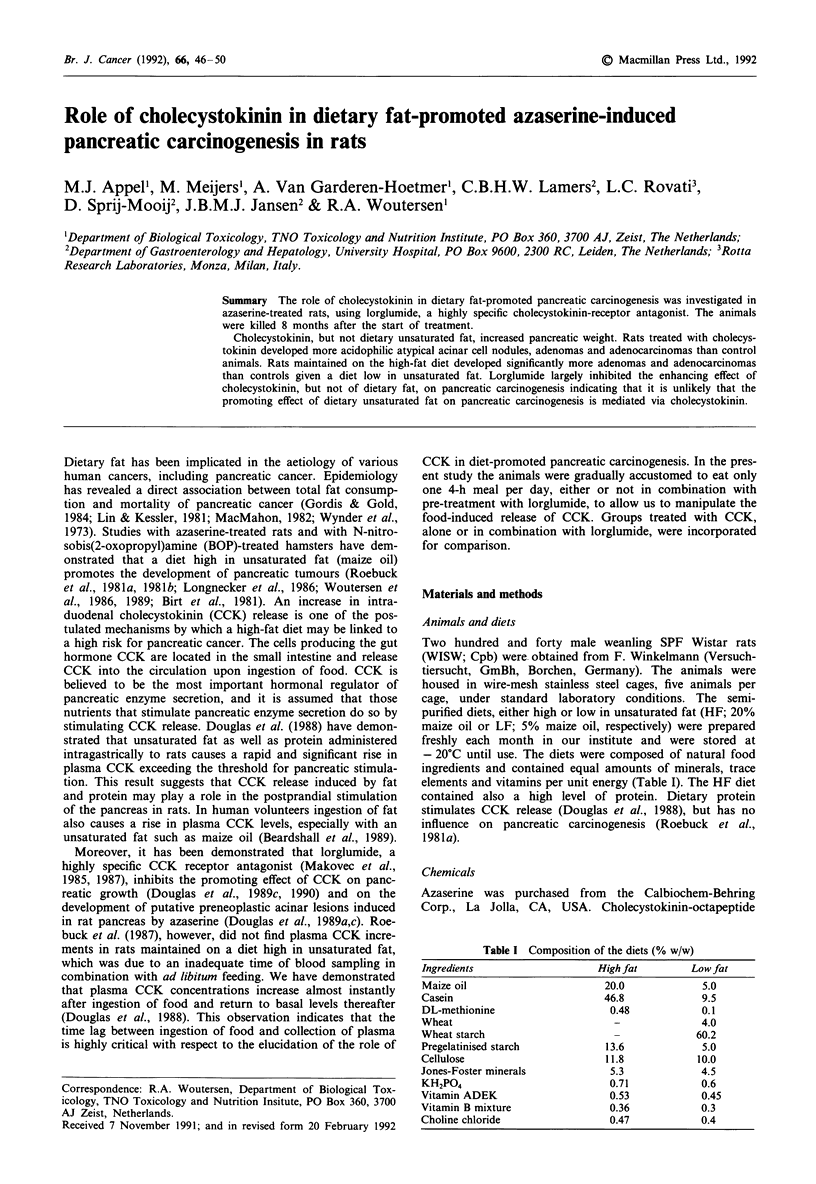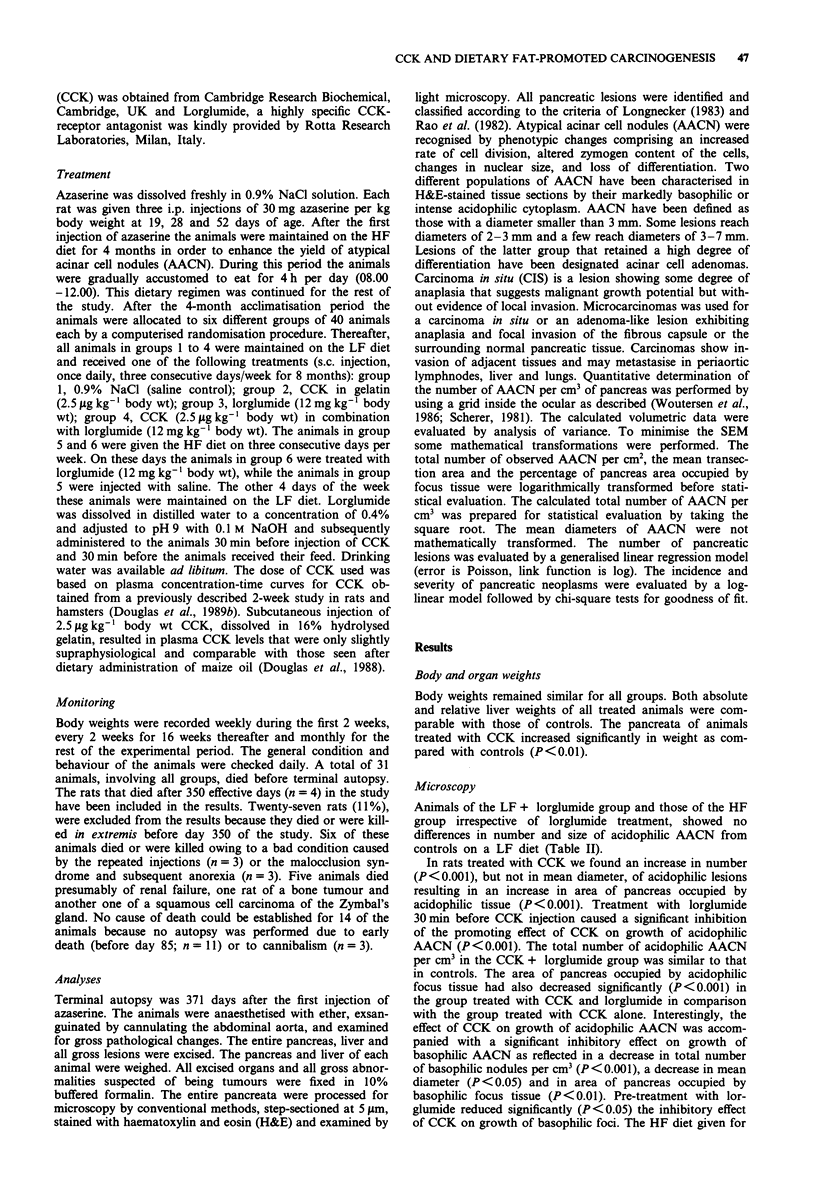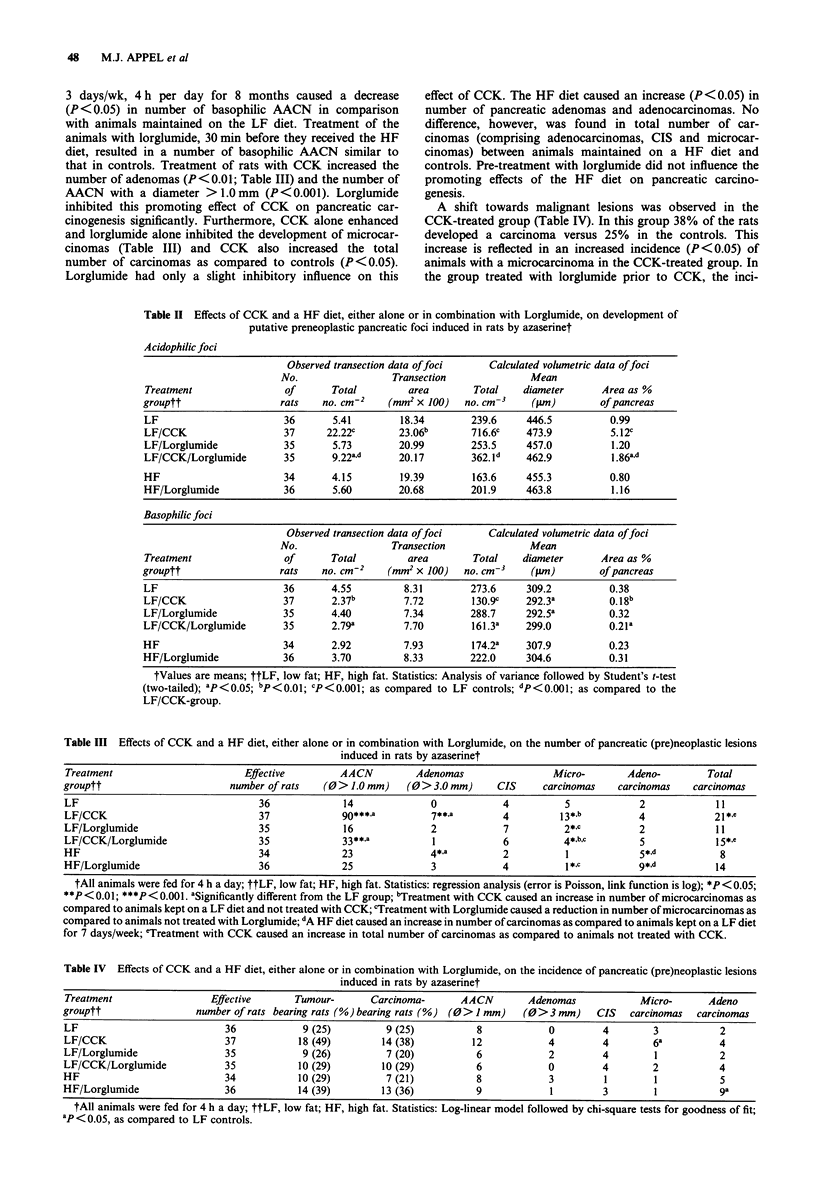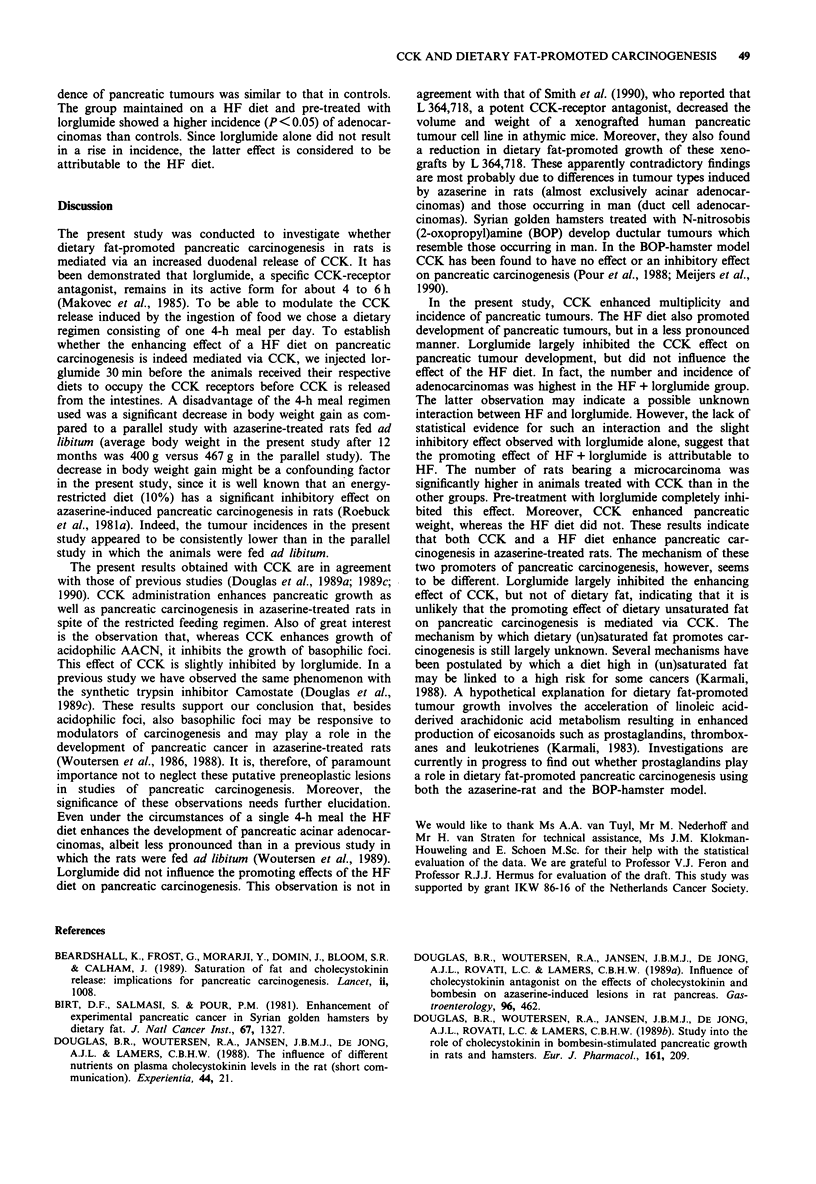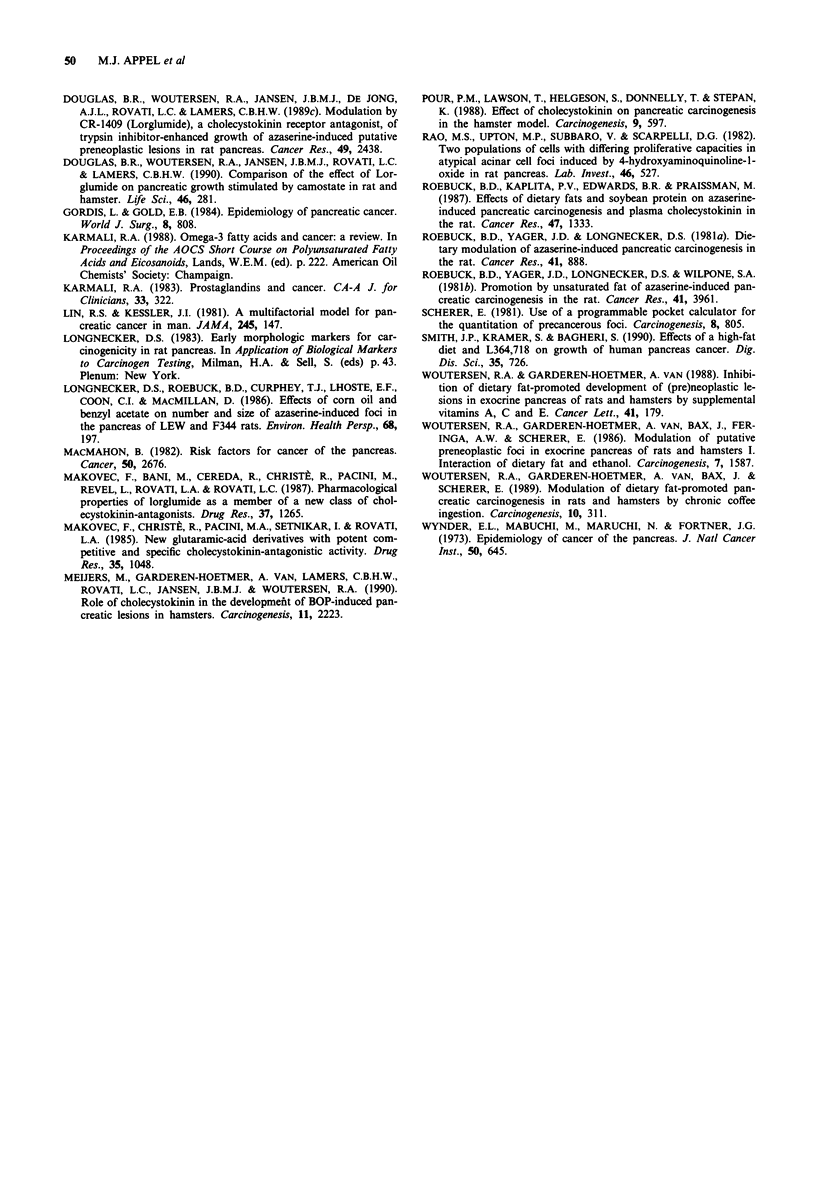# Role of cholecystokinin in dietary fat-promoted azaserine-induced pancreatic carcinogenesis in rats.

**DOI:** 10.1038/bjc.1992.214

**Published:** 1992-07

**Authors:** M. J. Appel, M. Meijers, A. Van Garderen-Hoetmer, C. B. Lamers, L. C. Rovati, D. Sprij-Mooij, J. B. Jansen, R. A. Woutersen

**Affiliations:** Department of Biological Toxicology, TNO Toxicology and Nutrition Institute, Zeist, The Netherlands.

## Abstract

The role of cholecystokinin in dietary fat-promoted pancreatic carcinogenesis was investigated in azaserine-treated rats, using lorglumide, a highly specific cholecystokinin-receptor antagonist. The animals were killed 8 months after the start of treatment. Cholecystokinin, but not dietary unsaturated fat, increased pancreatic weight. Rats treated with cholecystokinin developed more acidophilic atypical acinar cell nodules, adenomas and adenocarcinomas than control animals. Rats maintained on the high-fat diet developed significantly more adenomas and adenocarcinomas than controls given a diet low in unsaturated fat. Lorglumide largely inhibited the enhancing effect of cholecystokinin, but not of dietary fat, on pancreatic carcinogenesis indicating that it is unlikely that the promoting effect of dietary unsaturated fat on pancreatic carcinogenesis is mediated via cholecystokinin.


*Br. J. Cancer* (1992), **66**, 46–50 © Macmillan Press Ltd., 1992

### Role of cholecystokinin in dietary fat-promoted azaserine-induced pancreatic carcinogenesis in rats

M.J. Appel[1], M. Meijers[1], A. Van Garderen-Hoetmer[1], C.B.H.W. Lamers[2], L.C. Rovati[3], D. Sprij-Mooij[2], J.B.M.J. Jansen[2] & R.A. Woutersen[1]

[1]*Department of Biological Toxicology, TNO Toxicology and Nutrition Institute, PO Box 360, 3700 AJ, Zeist, The Netherlands;* [2]*Department of Gastroenterology and Hepatology, University Hospital, PO Box 9600, 2300 RC, Leiden, The Netherlands;* [3]*Rotta Research Laboratories, Monza, Milan, Italy.*

**Summary** The role of cholecystokinin in dietary fat-promoted pancreatic carcinogenesis was investigated in azaserine-treated rats, using lorglumide, a highly specific cholecystokinin-receptor antagonist. The animals were killed 8 months after the start of treatment.

Cholecystokinin, but not dietary unsaturated fat, increased pancreatic weight. Rats treated with cholecystokinin developed more acidophilic atypical acinar cell nodules, adenomas and adenocarcinomas than control animals. Rats maintained on the high-fat diet developed significantly more adenomas and adenocarcinomas than controls given a diet low in unsaturated fat. Lorglumide largely inhibited the enhancing effect of cholecystokinin, but not of dietary fat, on pancreatic carcinogenesis indicating that it is unlikely that the promoting effect of dietary unsaturated fat on pancreatic carcinogenesis is mediated via cholecystokinin.

Dietary fat has been implicated in the aetiology of various human cancers, including pancreatic cancer. Epidemiology has revealed a direct association between total fat consumption and mortality of pancreatic cancer (Gordis & Gold, 1984; Lin & Kessler, 1981; MacMahon, 1982; Wynder *et al.*, 1973). Studies with azaserine-treated rats and with N-nitrosobis(2-oxopropyl)amine (BOP)-treated hamsters have demonstrated that a diet high in unsaturated fat (maize oil) promotes the development of pancreatic tumours (Roebuck *et al.*, 1981*a*, 1981*b*; Longnecker *et al.*, 1986; Woutersen *et al.*, 1986, 1989; Birt *et al.*, 1981). An increase in intraduodenal cholecystokinin (CCK) release is one of the postulated mechanisms by which a high-fat diet may be linked to a high risk for pancreatic cancer. The cells producing the gut hormone CCK are located in the small intestine and release CCK into the circulation upon ingestion of food. CCK is believed to be the most important hormonal regulator of pancreatic enzyme secretion, and it is assumed that those nutrients that stimulate pancreatic enzyme secretion do so by stimulating CCK release. Douglas *et al.* (1988) have demonstrated that unsaturated fat as well as protein administered intragastrically to rats causes a rapid and significant rise in plasma CCK exceeding the threshold for pancreatic stimulation. This result suggests that CCK release induced by fat and protein may play a role in the postprandial stimulation of the pancreas in rats. In human volunteers ingestion of fat also causes a rise in plasma CCK levels, especially with an unsaturated fat such as maize oil (Beardshall *et al.*, 1989).

Moreover, it has been demonstrated that lorglumide, a highly specific CCK receptor antagonist (Makovec *et al.*, 1985, 1987), inhibits the promoting effect of CCK on pancreatic growth (Douglas *et al.*, 1989*c*, 1990) and on the development of putative preneoplastic acinar lesions induced in rat pancreas by azaserine (Douglas *et al.*, 1989*a,c*). Roebuck *et al.* (1987), however, did not find plasma CCK increments in rats maintained on a diet high in unsaturated fat, which was due to an inadequate time of blood sampling in combination with *ad libitum* feeding. We have demonstrated that plasma CCK concentrations increase almost instantly after ingestion of food and return to basal levels thereafter (Douglas *et al.*, 1988). This observation indicates that the time lag between ingestion of food and collection of plasma is highly critical with respect to the elucidation of the role of CCK in diet-promoted pancreatic carcinogenesis. In the present study the animals were gradually accustomed to eat only one 4-h meal per day, either or not in combination with pre-treatment with lorglumide, to allow us to manipulate the food-induced release of CCK. Groups treated with CCK, alone or in combination with lorglumide, were incorporated for comparison.

Correspondence: R.A. Woutersen, Department of Biological Toxicology, TNO Toxicology and Nutrition Insitute, PO Box 360, 3700 AJ Zeist, Netherlands.
Received 7 November 1991; and in revised form 20 February 1992

### Materials and methods

### *Animals and diets*

Two hundred and forty male weanling SPF Wistar rats (WISW; Cpb) were obtained from F. Winkelmann (Versuchtiersucht, GmBh, Borchen, Germany). The animals were housed in wire-mesh stainless steel cages, five animals per cage, under standard laboratory conditions. The semipurified diets, either high or low in unsaturated fat (HF; 20% maize oil or LF; 5% maize oil, respectively) were prepared freshly each month in our institute and were stored at − 20°C until use. The diets were composed of natural food ingredients and contained equal amounts of minerals, trace elements and vitamins per unit energy (Table I). The HF diet contained also a high level of protein. Dietary protein stimulates CCK release (Douglas *et al.*, 1988), but has no influence on pancreatic carcinogenesis (Roebuck *et al.*, 1981*a*).

### *Chemicals*

Azaserine was purchased from the Calbiochem-Behring Corp., La Jolla, CA, USA. Cholecystokinin-octapeptide

**Table I** Composition of the diets (% w/w)

| *Ingredients* | *High fat* | *Low fat* |
|---|---|---|
| Maize oil | 20.0 | 5.0 |
| Casein | 46.8 | 9.5 |
| DL-methionine | 0.48 | 0.1 |
| Wheat | – | 4.0 |
| Wheat starch | – | 60.2 |
| Pregelatinised starch | 13.6 | 5.0 |
| Cellulose | 11.8 | 10.0 |
| Jones-Foster minerals | 5.3 | 4.5 |
| $KH_2PO_4$ | 0.71 | 0.6 |
| Vitamin ADEK | 0.53 | 0.45 |
| Vitamin B mixture | 0.36 | 0.3 |
| Choline chloride | 0.47 | 0.4 |

(CCK) was obtained from Cambridge Research Biochemical, Cambridge, UK and Lorglumide, a highly specific CCK-receptor antagonist was kindly provided by Rotta Research Laboratories, Milan, Italy.

*Treatment*

Azaserine was dissolved freshly in 0.9% NaCl solution. Each rat was given three i.p. injections of 30 mg azaserine per kg body weight at 19, 28 and 52 days of age. After the first injection of azaserine the animals were maintained on the HF diet for 4 months in order to enhance the yield of atypical acinar cell nodules (AACN). During this period the animals were gradually accustomed to eat for 4 h per day (08.00 – 12.00). This dietary regimen was continued for the rest of the study. After the 4-month acclimatisation period the animals were allocated to six different groups of 40 animals each by a computerised randomisation procedure. Thereafter, all animals in groups 1 to 4 were maintained on the LF diet and received one of the following treatments (s.c. injection, once daily, three consecutive days/week for 8 months): group 1, 0.9% NaCl (saline control); group 2, CCK in gelatin (2.5 $\mu g\ kg^{-1}$ body wt); group 3, lorglumide (12 $mg\ kg^{-1}$ body wt); group 4, CCK (2.5 $\mu g\ kg^{-1}$ body wt) in combination with lorglumide (12 $mg\ kg^{-1}$ body wt). The animals in group 5 and 6 were given the HF diet on three consecutive days per week. On these days the animals in group 6 were treated with lorglumide (12 $mg\ kg^{-1}$ body wt), while the animals in group 5 were injected with saline. The other 4 days of the week these animals were maintained on the LF diet. Lorglumide was dissolved in distilled water to a concentration of 0.4% and adjusted to pH 9 with 0.1 M NaOH and subsequently administered to the animals 30 min before injection of CCK and 30 min before the animals received their feed. Drinking water was available *ad libitum*. The dose of CCK used was based on plasma concentration-time curves for CCK obtained from a previously described 2-week study in rats and hamsters (Douglas *et al.*, 1989*b*). Subcutaneous injection of 2.5 $\mu g\ kg^{-1}$ body wt CCK, dissolved in 16% hydrolysed gelatin, resulted in plasma CCK levels that were only slightly supraphysiological and comparable with those seen after dietary administration of maize oil (Douglas *et al.*, 1988).

*Monitoring*

Body weights were recorded weekly during the first 2 weeks, every 2 weeks for 16 weeks thereafter and monthly for the rest of the experimental period. The general condition and behaviour of the animals were checked daily. A total of 31 animals, involving all groups, died before terminal autopsy. The rats that died after 350 effective days ($n = 4$) in the study have been included in the results. Twenty-seven rats (11%), were excluded from the results because they died or were killed *in extremis* before day 350 of the study. Six of these animals died or were killed owing to a bad condition caused by the repeated injections ($n = 3$) or the malocclusion syndrome and subsequent anorexia ($n = 3$). Five animals died presumably of renal failure, one rat of a bone tumour and another one of a squamous cell carcinoma of the Zymbal's gland. No cause of death could be established for 14 of the animals because no autopsy was performed due to early death (before day 85; $n = 11$) or to cannibalism ($n = 3$).

*Analyses*

Terminal autopsy was 371 days after the first injection of azaserine. The animals were anaesthetised with ether, exsanguinated by cannulating the abdominal aorta, and examined for gross pathological changes. The entire pancreas, liver and all gross lesions were excised. The pancreas and liver of each animal were weighed. All excised organs and all gross abnormalities suspected of being tumours were fixed in 10% buffered formalin. The entire pancreata were processed for microscopy by conventional methods, step-sectioned at 5 $\mu m$, stained with haematoxylin and eosin (H&E) and examined by light microscopy. All pancreatic lesions were identified and classified according to the criteria of Longnecker (1983) and Rao *et al.* (1982). Atypical acinar cell nodules (AACN) were recognised by phenotypic changes comprising an increased rate of cell division, altered zymogen content of the cells, changes in nuclear size, and loss of differentiation. Two different populations of AACN have been characterised in H&E-stained tissue sections by their markedly basophilic or intense acidophilic cytoplasm. AACN have been defined as those with a diameter smaller than 3 mm. Some lesions reach diameters of 2–3 mm and a few reach diameters of 3–7 mm. Lesions of the latter group that retained a high degree of differentiation have been designated acinar cell adenomas. Carcinoma *in situ* (CIS) is a lesion showing some degree of anaplasia that suggests malignant growth potential but without evidence of local invasion. Microcarcinomas was used for a carcinoma *in situ* or an adenoma-like lesion exhibiting anaplasia and focal invasion of the fibrous capsule or the surrounding normal pancreatic tissue. Carcinomas show invasion of adjacent tissues and may metastasise in periaortic lymphnodes, liver and lungs. Quantitative determination of the number of AACN per $cm^3$ of pancreas was performed by using a grid inside the ocular as described (Woutersen *et al.*, 1986; Scherer, 1981). The calculated volumetric data were evaluated by analysis of variance. To minimise the SEM some mathematical transformations were performed. The total number of observed AACN per $cm^2$, the mean transection area and the percentage of pancreas area occupied by focus tissue were logarithmically transformed before statistical evaluation. The calculated total number of AACN per $cm^3$ was prepared for statistical evaluation by taking the square root. The mean diameters of AACN were not mathematically transformed. The number of pancreatic lesions was evaluated by a generalised linear regression model (error is Poisson, link function is log). The incidence and severity of pancreatic neoplasms were evaluated by a log-linear model followed by chi-square tests for goodness of fit.

### Results

*Body and organ weights*

Body weights remained similar for all groups. Both absolute and relative liver weights of all treated animals were comparable with those of controls. The pancreata of animals treated with CCK increased significantly in weight as compared with controls ($P < 0.01$).

*Microscopy*

Animals of the LF + lorglumide group and those of the HF group irrespective of lorglumide treatment, showed no differences in number and size of acidophilic AACN from controls on a LF diet (Table II).

In rats treated with CCK we found an increase in number ($P < 0.001$), but not in mean diameter, of acidophilic lesions resulting in an increase in area of pancreas occupied by acidophilic tissue ($P < 0.001$). Treatment with lorglumide 30 min before CCK injection caused a significant inhibition of the promoting effect of CCK on growth of acidophilic AACN ($P < 0.001$). The total number of acidophilic AACN per $cm^3$ in the CCK + lorglumide group was similar to that in controls. The area of pancreas occupied by acidophilic focus tissue had also decreased significantly ($P < 0.001$) in the group treated with CCK and lorglumide in comparison with the group treated with CCK alone. Interestingly, the effect of CCK on growth of acidophilic AACN was accompanied with a significant inhibitory effect on growth of basophilic AACN as reflected in a decrease in total number of basophilic nodules per $cm^3$ ($P < 0.001$), a decrease in mean diameter ($P < 0.05$) and in area of pancreas occupied by basophilic focus tissue ($P < 0.01$). Pre-treatment with lorglumide reduced significantly ($P < 0.05$) the inhibitory effect of CCK on growth of basophilic foci. The HF diet given for

3 days/wk, 4 h per day for 8 months caused a decrease ($P<0.05$) in number of basophilic AACN in comparison with animals maintained on the LF diet. Treatment of the animals with lorglumide, 30 min before they received the HF diet, resulted in a number of basophilic AACN similar to that in controls. Treatment of rats with CCK increased the number of adenomas ($P<0.01$; Table III) and the number of AACN with a diameter >1.0 mm ($P<0.001$). Lorglumide inhibited this promoting effect of CCK on pancreatic carcinogenesis significantly. Furthermore, CCK alone enhanced and lorglumide alone inhibited the development of microcarcinomas (Table III) and CCK also increased the total number of carcinomas as compared to controls ($P<0.05$). Lorglumide had only a slight inhibitory influence on this effect of CCK. The HF diet caused an increase ($P<0.05$) in number of pancreatic adenomas and adenocarcinomas. No difference, however, was found in total number of carcinomas (comprising adenocarcinomas, CIS and microcarcinomas) between animals maintained on a HF diet and controls. Pre-treatment with lorglumide did not influence the promoting effects of the HF diet on pancreatic carcinogenesis.

A shift towards malignant lesions was observed in the CCK-treated group (Table IV). In this group 38% of the rats developed a carcinoma versus 25% in the controls. This increase is reflected in an increased incidence ($P<0.05$) of animals with a microcarcinoma in the CCK-treated group. In the group treated with lorglumide prior to CCK, the inci-

**Table II** Effects of CCK and a HF diet, either alone or in combination with Lorglumide, on development of putative preneoplastic pancreatic foci induced in rats by azaserine†

*Acidophilic foci*

| Treatment group†† | Observed transection data of foci: No. of rats | Total no. $cm^{-2}$ | Transection area ($mm^2 \times 100$) | Calculated volumetric data of foci: Total no. $cm^{-3}$ | Mean diameter (μm) | Area as % of pancreas |
|---|---|---|---|---|---|---|
| LF | 36 | 5.41 | 18.34 | 239.6 | 446.5 | 0.99 |
| LF/CCK | 37 | 22.22[c] | 23.06[b] | 716.6[c] | 473.9 | 5.12[c] |
| LF/Lorglumide | 35 | 5.73 | 20.99 | 253.5 | 457.0 | 1.20 |
| LF/CCK/Lorglumide | 35 | 9.22[a,d] | 20.17 | 362.1[d] | 462.9 | 1.86[a,d] |
| HF | 34 | 4.15 | 19.39 | 163.6 | 455.3 | 0.80 |
| HF/Lorglumide | 36 | 5.60 | 20.68 | 201.9 | 463.8 | 1.16 |

*Basophilic foci*

| Treatment group†† | Observed transection data of foci: No. of rats | Total no. $cm^{-2}$ | Transection area ($mm^2 \times 100$) | Calculated volumetric data of foci: Total no. $cm^{-3}$ | Mean diameter (μm) | Area as % of pancreas |
|---|---|---|---|---|---|---|
| LF | 36 | 4.55 | 8.31 | 273.6 | 309.2 | 0.38 |
| LF/CCK | 37 | 2.37[b] | 7.72 | 130.9[c] | 292.3[a] | 0.18[b] |
| LF/Lorglumide | 35 | 4.40 | 7.34 | 288.7 | 292.5[a] | 0.32 |
| LF/CCK/Lorglumide | 35 | 2.79[a] | 7.70 | 161.3[a] | 299.0 | 0.21[a] |
| HF | 34 | 2.92 | 7.93 | 174.2[a] | 307.9 | 0.23 |
| HF/Lorglumide | 36 | 3.70 | 8.33 | 222.0 | 304.6 | 0.31 |

†Values are means; ††LF, low fat; HF, high fat. Statistics: Analysis of variance followed by Student's $t$-test (two-tailed); [a]$P<0.05$; [b]$P<0.01$; [c]$P<0.001$; as compared to LF controls; [d]$P<0.001$; as compared to the LF/CCK-group.

**Table III** Effects of CCK and a HF diet, either alone or in combination with Lorglumide, on the number of pancreatic (pre)neoplastic lesions induced in rats by azaserine†

| Treatment group†† | Effective number of rats | AACN (Ø>1.0 mm) | Adenomas (Ø>3.0 mm) | CIS | Micro-carcinomas | Adeno-carcinomas | Total carcinomas |
|---|---|---|---|---|---|---|---|
| LF | 36 | 14 | 0 | 4 | 5 | 2 | 11 |
| LF/CCK | 37 | 90***,a | 7**,a | 4 | 13*,b | 4 | 21*,e |
| LF/Lorglumide | 35 | 16 | 2 | 7 | 2*,c | 2 | 11 |
| LF/CCK/Lorglumide | 35 | 33**,a | 1 | 6 | 4*,b,c | 5 | 15*,e |
| HF | 34 | 23 | 4*,a | 2 | 1 | 5*,d | 8 |
| HF/Lorglumide | 36 | 25 | 3 | 4 | 1*,c | 9*,d | 14 |

†All animals were fed for 4 h a day; ††LF, low fat; HF, high fat. Statistics: regression analysis (error is Poisson, link function is log); *$P<0.05$; **$P<0.01$; ***$P<0.001$. [a]Significantly different from the LF group; [b]Treatment with CCK caused an increase in number of microcarcinomas as compared to animals kept on a LF diet and not treated with CCK; [c]Treatment with Lorglumide caused a reduction in number of microcarcinomas as compared to animals not treated with Lorglumide; [d]A HF diet caused an increase in number of carcinomas as compared to animals kept on a LF diet for 7 days/week; [e]Treatment with CCK caused an increase in total number of carcinomas as compared to animals not treated with CCK.

**Table IV** Effects of CCK and a HF diet, either alone or in combination with Lorglumide, on the incidence of pancreatic (pre)neoplastic lesions induced in rats by azaserine†

| Treatment group†† | Effective number of rats | Tumour-bearing rats (%) | Carcinoma-bearing rats (%) | AACN (Ø>1 mm) | Adenomas (Ø>3 mm) | CIS | Micro-carcinomas | Adeno carcinomas |
|---|---|---|---|---|---|---|---|---|
| LF | 36 | 9 (25) | 9 (25) | 8 | 0 | 4 | 3 | 2 |
| LF/CCK | 37 | 18 (49) | 14 (38) | 12 | 4 | 4 | 6[a] | 4 |
| LF/Lorglumide | 35 | 9 (26) | 7 (20) | 6 | 2 | 4 | 1 | 2 |
| LF/CCK/Lorglumide | 35 | 10 (29) | 10 (29) | 6 | 0 | 4 | 2 | 4 |
| HF | 34 | 10 (29) | 7 (21) | 8 | 3 | 1 | 1 | 5 |
| HF/Lorglumide | 36 | 14 (39) | 13 (36) | 9 | 1 | 3 | 1 | 9[a] |

†All animals were fed for 4 h a day; ††LF, low fat; HF, high fat. Statistics: Log-linear model followed by chi-square tests for goodness of fit; [a]$P<0.05$, as compared to LF controls.

dence of pancreatic tumours was similar to that in controls. The group maintained on a HF diet and pre-treated with lorglumide showed a higher incidence ($P < 0.05$) of adenocarcinomas than controls. Since lorglumide alone did not result in a rise in incidence, the latter effect is considered to be attributable to the HF diet.

### Discussion

The present study was conducted to investigate whether dietary fat-promoted pancreatic carcinogenesis in rats is mediated via an increased duodenal release of CCK. It has been demonstrated that lorglumide, a specific CCK-receptor antagonist, remains in its active form for about 4 to 6 h (Makovec *et al.*, 1985). To be able to modulate the CCK release induced by the ingestion of food we chose a dietary regimen consisting of one 4-h meal per day. To establish whether the enhancing effect of a HF diet on pancreatic carcinogenesis is indeed mediated via CCK, we injected lorglumide 30 min before the animals received their respective diets to occupy the CCK receptors before CCK is released from the intestines. A disadvantage of the 4-h meal regimen used was a significant decrease in body weight gain as compared to a parallel study with azaserine-treated rats fed *ad libitum* (average body weight in the present study after 12 months was 400 g versus 467 g in the parallel study). The decrease in body weight gain might be a confounding factor in the present study, since it is well known that an energy-restricted diet (10%) has a significant inhibitory effect on azaserine-induced pancreatic carcinogenesis in rats (Roebuck *et al.*, 1981*a*). Indeed, the tumour incidences in the present study appeared to be consistently lower than in the parallel study in which the animals were fed *ad libitum*.

The present results obtained with CCK are in agreement with those of previous studies (Douglas *et al.*, 1989*a*; 1989*c*; 1990). CCK administration enhances pancreatic growth as well as pancreatic carcinogenesis in azaserine-treated rats in spite of the restricted feeding regimen. Also of great interest is the observation that, whereas CCK enhances growth of acidophilic AACN, it inhibits the growth of basophilic foci. This effect of CCK is slightly inhibited by lorglumide. In a previous study we have observed the same phenomenon with the synthetic trypsin inhibitor Camostate (Douglas *et al.*, 1989*c*). These results support our conclusion that, besides acidophilic foci, also basophilic foci may be responsive to modulators of carcinogenesis and may play a role in the development of pancreatic cancer in azaserine-treated rats (Woutersen *et al.*, 1986, 1988). It is, therefore, of paramount importance not to neglect these putative preneoplastic lesions in studies of pancreatic carcinogenesis. Moreover, the significance of these observations needs further elucidation. Even under the circumstances of a single 4-h meal the HF diet enhances the development of pancreatic acinar adenocarcinomas, albeit less pronounced than in a previous study in which the rats were fed *ad libitum* (Woutersen *et al.*, 1989). Lorglumide did not influence the promoting effects of the HF diet on pancreatic carcinogenesis. This observation is not in agreement with that of Smith *et al.* (1990), who reported that L 364,718, a potent CCK-receptor antagonist, decreased the volume and weight of a xenografted human pancreatic tumour cell line in athymic mice. Moreover, they also found a reduction in dietary fat-promoted growth of these xenografts by L 364,718. These apparently contradictory findings are most probably due to differences in tumour types induced by azaserine in rats (almost exclusively acinar adenocarcinomas) and those occurring in man (duct cell adenocarcinomas). Syrian golden hamsters treated with N-nitrosobis (2-oxopropyl)amine (BOP) develop ductular tumours which resemble those occurring in man. In the BOP-hamster model CCK has been found to have no effect or an inhibitory effect on pancreatic carcinogenesis (Pour *et al.*, 1988; Meijers *et al.*, 1990).

In the present study, CCK enhanced multiplicity and incidence of pancreatic tumours. The HF diet also promoted development of pancreatic tumours, but in a less pronounced manner. Lorglumide largely inhibited the CCK effect on pancreatic tumour development, but did not influence the effect of the HF diet. In fact, the number and incidence of adenocarcinomas was highest in the HF + lorglumide group. The latter observation may indicate a possible unknown interaction between HF and lorglumide. However, the lack of statistical evidence for such an interaction and the slight inhibitory effect observed with lorglumide alone, suggest that the promoting effect of HF + lorglumide is attributable to HF. The number of rats bearing a microcarcinoma was significantly higher in animals treated with CCK than in the other groups. Pre-treatment with lorglumide completely inhibited this effect. Moreover, CCK enhanced pancreatic weight, whereas the HF diet did not. These results indicate that both CCK and a HF diet enhance pancreatic carcinogenesis in azaserine-treated rats. The mechanism of these two promoters of pancreatic carcinogenesis, however, seems to be different. Lorglumide largely inhibited the enhancing effect of CCK, but not of dietary fat, indicating that it is unlikely that the promoting effect of dietary unsaturated fat on pancreatic carcinogenesis is mediated via CCK. The mechanism by which dietary (un)saturated fat promotes carcinogenesis is still largely unknown. Several mechanisms have been postulated by which a diet high in (un)saturated fat may be linked to a high risk for some cancers (Karmali, 1988). A hypothetical explanation for dietary fat-promoted tumour growth involves the acceleration of linoleic acid-derived arachidonic acid metabolism resulting in enhanced production of eicosanoids such as prostaglandins, thromboxanes and leukotrienes (Karmali, 1983). Investigations are currently in progress to find out whether prostaglandins play a role in dietary fat-promoted pancreatic carcinogenesis using both the azaserine-rat and the BOP-hamster model.

We would like to thank Ms A.A. van Tuyl, Mr M. Nederhoff and Mr H. van Straten for technical assistance, Ms J.M. Klokman-Houweling and E. Schoen M.Sc. for their help with the statistical evaluation of the data. We are grateful to Professor V.J. Feron and Professor R.J.J. Hermus for evaluation of the draft. This study was supported by grant IKW 86-16 of the Netherlands Cancer Society.

### References

BEARDSHALL, K., FROST, G., MORARJI, Y., DOMIN, J., BLOOM, S.R. & CALHAM, J. (1989). Saturation of fat and cholecystokinin release: implications for pancreatic carcinogenesis. *Lancet*, **ii**, 1008.

BIRT, D.F., SALMASI, S. & POUR, P.M. (1981). Enhancement of experimental pancreatic cancer in Syrian golden hamsters by dietary fat. *J. Natl Cancer Inst.*, **67**, 1327.

DOUGLAS, B.R., WOUTERSEN, R.A., JANSEN, J.B.M.J., DE JONG, A.J.L. & LAMERS, C.B.H.W. (1988). The influence of different nutrients on plasma cholecystokinin levels in the rat (short communication). *Experientia*, **44**, 21.

DOUGLAS, B.R., WOUTERSEN, R.A., JANSEN, J.B.M.J., DE JONG, A.J.L., ROVATI, L.C. & LAMERS, C.B.H.W. (1989*a*). Influence of cholecystokinin antagonist on the effects of cholecystokinin and bombesin on azaserine-induced lesions in rat pancreas. *Gastroenterology*, **96**, 462.

DOUGLAS, B.R., WOUTERSEN, R.A., JANSEN, J.B.M.J., DE JONG, A.J.L., ROVATI, L.C. & LAMERS, C.B.H.W. (1989*b*). Study into the role of cholecystokinin in bombesin-stimulated pancreatic growth in rats and hamsters. *Eur. J. Pharmacol.*, **161**, 209.

DOUGLAS, B.R., WOUTERSEN, R.A., JANSEN, J.B.M.J., DE JONG, A.J.L., ROVATI, L.C. & LAMERS, C.B.H.W. (1989*c*). Modulation by CR-1409 (Lorglumide), a cholecystokinin receptor antagonist, of trypsin inhibitor-enhanced growth of azaserine-induced putative preneoplastic lesions in rat pancreas. *Cancer Res.*, **49**, 2438.

DOUGLAS, B.R., WOUTERSEN, R.A., JANSEN, J.B.M.J., ROVATI, L.C. & LAMERS, C.B.H.W. (1990). Comparison of the effect of Lorglumide on pancreatic growth stimulated by camostate in rat and hamster. *Life Sci.*, **46**, 281.

GORDIS, L. & GOLD, E.B. (1984). Epidemiology of pancreatic cancer. *World J. Surg.*, **8**, 808.

KARMALI, R.A. (1988). Omega-3 fatty acids and cancer: a review. In *Proceedings of the AOCS Short Course on Polyunsaturated Fatty Acids and Eicosanoids*, Lands, W.E.M. (ed). p. 222. American Oil Chemists' Society: Champaign.

KARMALI, R.A. (1983). Prostaglandins and cancer. *CA-A J. for Clinicians*, **33**, 322.

LIN, R.S. & KESSLER, J.I. (1981). A multifactorial model for pancreatic cancer in man. *JAMA*, **245**, 147.

LONGNECKER, D.S. (1983). Early morphologic markers for carcinogenicity in rat pancreas. In *Application of Biological Markers to Carcinogen Testing*, Milman, H.A. & Sell, S. (eds) p. 43. Plenum: New York.

LONGNECKER, D.S., ROEBUCK, B.D., CURPHEY, T.J., LHOSTE, E.F., COON, C.I. & MACMILLAN, D. (1986). Effects of corn oil and benzyl acetate on number and size of azaserine-induced foci in the pancreas of LEW and F344 rats. *Environ. Health Persp.*, **68**, 197.

MACMAHON, B. (1982). Risk factors for cancer of the pancreas. *Cancer*, **50**, 2676.

MAKOVEC, F., BANI, M., CEREDA, R., CHRISTÈ, R., PACINI, M., REVEL, L., ROVATI, L.A. & ROVATI, L.C. (1987). Pharmacological properties of lorglumide as a member of a new class of cholecystokinin-antagonists. *Drug Res.*, **37**, 1265.

MAKOVEC, F., CHRISTÈ, R., PACINI, M.A., SETNIKAR, I. & ROVATI, L.A. (1985). New glutaramic-acid derivatives with potent competitive and specific cholecystokinin-antagonistic activity. *Drug Res.*, **35**, 1048.

MEIJERS, M., GARDEREN-HOETMER, A. VAN, LAMERS, C.B.H.W., ROVATI, L.C., JANSEN, J.B.M.J. & WOUTERSEN, R.A. (1990). Role of cholecystokinin in the development of BOP-induced pancreatic lesions in hamsters. *Carcinogenesis*, **11**, 2223.

POUR, P.M., LAWSON, T., HELGESON, S., DONNELLY, T. & STEPAN, K. (1988). Effect of cholecystokinin on pancreatic carcinogenesis in the hamster model. *Carcinogenesis*, **9**, 597.

RAO, M.S., UPTON, M.P., SUBBARO, V. & SCARPELLI, D.G. (1982). Two populations of cells with differing proliferative capacities in atypical acinar cell foci induced by 4-hydroxyaminoquinoline-1-oxide in rat pancreas. *Lab. Invest.*, **46**, 527.

ROEBUCK, B.D., KAPLITA, P.V., EDWARDS, B.R. & PRAISSMAN, M. (1987). Effects of dietary fats and soybean protein on azaserine-induced pancreatic carcinogenesis and plasma cholecystokinin in the rat. *Cancer Res.*, **47**, 1333.

ROEBUCK, B.D., YAGER, J.D. & LONGNECKER, D.S. (1981*a*). Dietary modulation of azaserine-induced pancreatic carcinogenesis in the rat. *Cancer Res.*, **41**, 888.

ROEBUCK, B.D., YAGER, J.D., LONGNECKER, D.S. & WILPONE, S.A. (1981*b*). Promotion by unsaturated fat of azaserine-induced pancreatic carcinogenesis in the rat. *Cancer Res.*, **41**, 3961.

SCHERER, E. (1981). Use of a programmable pocket calculator for the quantitation of precancerous foci. *Carcinogenesis*, **8**, 805.

SMITH, J.P., KRAMER, S. & BAGHERI, S. (1990). Effects of a high-fat diet and L364,718 on growth of human pancreas cancer. *Dig. Dis. Sci.*, **35**, 726.

WOUTERSEN, R.A. & GARDEREN-HOETMER, A. VAN (1988). Inhibition of dietary fat-promoted development of (pre)neoplastic lesions in exocrine pancreas of rats and hamsters by supplemental vitamins A, C and E. *Cancer Lett.*, **41**, 179.

WOUTERSEN, R.A., GARDEREN-HOETMER, A. VAN, BAX, J., FERINGA, A.W. & SCHERER, E. (1986). Modulation of putative preneoplastic foci in exocrine pancreas of rats and hamsters I. Interaction of dietary fat and ethanol. *Carcinogenesis*, **7**, 1587.

WOUTERSEN, R.A., GARDEREN-HOETMER, A. VAN, BAX, J. & SCHERER, E. (1989). Modulation of dietary fat-promoted pancreatic carcinogenesis in rats and hamsters by chronic coffee ingestion. *Carcinogenesis*, **10**, 311.

WYNDER, E.L., MABUCHI, M., MARUCHI, N. & FORTNER, J.G. (1973). Epidemiology of cancer of the pancreas. *J. Natl Cancer Inst.*, **50**, 645.